# Tension-Free Management of Surgical Wound in Paramalleolar Bypass

**DOI:** 10.3400/avd.oa.20-00064

**Published:** 2020-09-25

**Authors:** Yoshihiko Tsuji, Ikuro Kitano, Yoriko Tsuji

**Affiliations:** 1 Department of Vascular Surgery, Shinsuma General Hospital; 2 Department of Plastic Surgery, Shinsuma General Hospital

**Keywords:** critical limb-threatening ischemia, paramalleolar bypass, wound management, artificial dermis

## Abstract

**Objective**: In paramalleolar bypass for critical limb-threatening ischemia (CLTI), excessive skin tension may occur for the closure of surgical wounds around the ankle. Furthermore, these surgical incisions are often proximal to infectious ischemic ulcers. Wound dehiscence caused by skin tension and surgical site infection carries a risk of graft exposure, anastomotic disruption, or graft insufficiency.

**Patients and Methods**: Tension-free wound management was adopted in eight patients who underwent paramalleolar bypass for CLTI. Tension-free closure was adopted for surgical incisions for distal anastomotic site of the paramalleolar bypass, whereas the incisions for saphenous vein harvest were left open. A relief incision was made as needed. The opened incisions were covered with artificial dermis.

**Results**: All surgical incisions and ischemic wounds healed successfully within 1.8 months after bypass. Two postoperative graft stenoses occurred, which were rescued by additional endovascular intervention. Secondary graft patency, wound healing, and limb salvage rates were 100% during an average follow-up period of 30 months.

**Conclusion**: Tension-free wound closure using artificial dermis was effective in selected cases of paramalleolar bypass for CLTI.

## Introduction

Paramalleolar bypass is a useful revascularization technique for treating critical limb-threatening ischemia (CLTI).^1–5)^ The reported outcomes of this bypass surgery appear to be acceptable, however, excellent patency rates and limb salvage rate of bypass surgery are veiled by the occurrence of wound complications.^1–19)^ In paramalleolar bypass, several incisions are made around the ankle where the skin is hard to extend; for exposure, from the dorsalis pedis artery to the pedal artery or from the posterior tibial artery to the plantar artery; and for harvesting the saphenous vein graft (SVG). Moreover, infectious ischemic ulcers are proximal to these surgical incisions and wound dehiscence caused by skin tension and surgical site infection has a risk of anastomotic disruption, graft thrombosis, and subsequent limb loss. Furthermore, the vein graft to the dorsalis pedis artery usually passes through the subcutaneous route and overrides in front of the tibia at the ankle level, and excessive skin tension induced by the vein graft passage promotes wound dehiscence and graft exposure.

In this study, we aimed to investigate the clinical outcomes of tension-free surgical wound management in paramalleolar bypass.

## Patients and Methods

**Figure 1:**
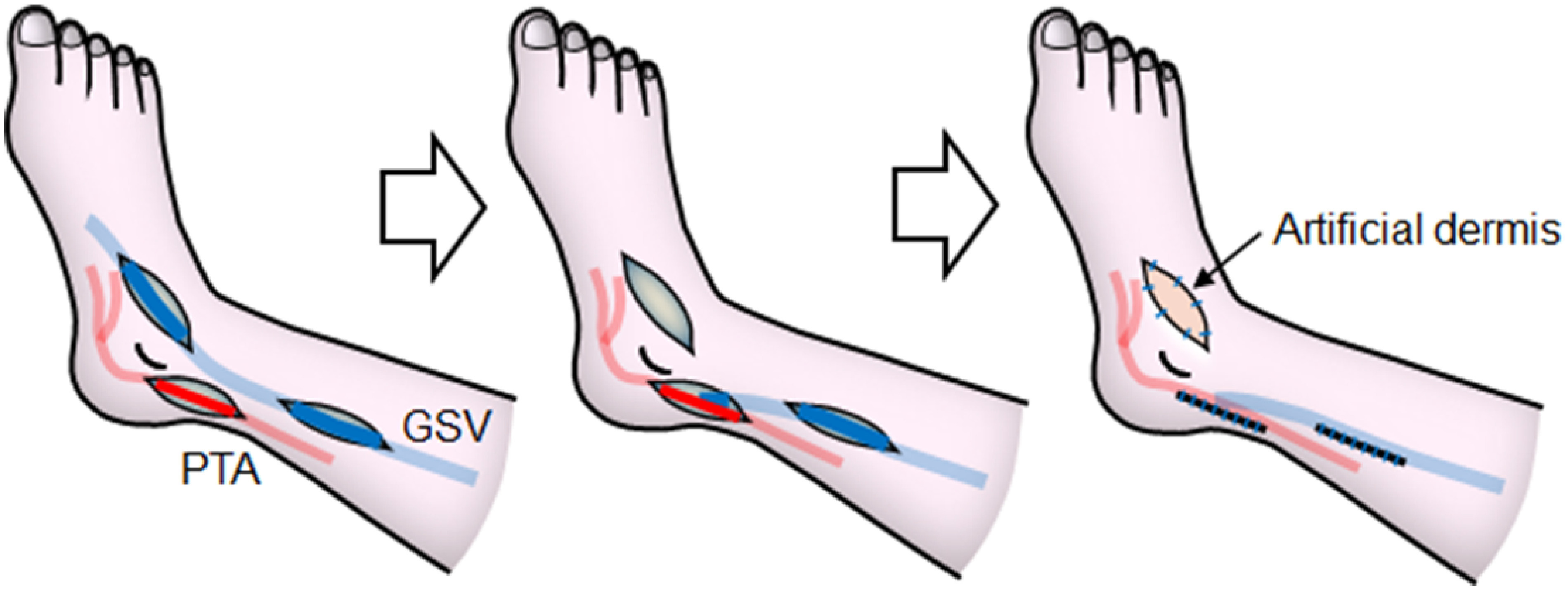
Fig. 1 Schema of the tension-free wound management in the bypass to the posterior tibial artery.

GSV: great saphenous vein; PTA: posterior tibial artery

**Figure 2:**
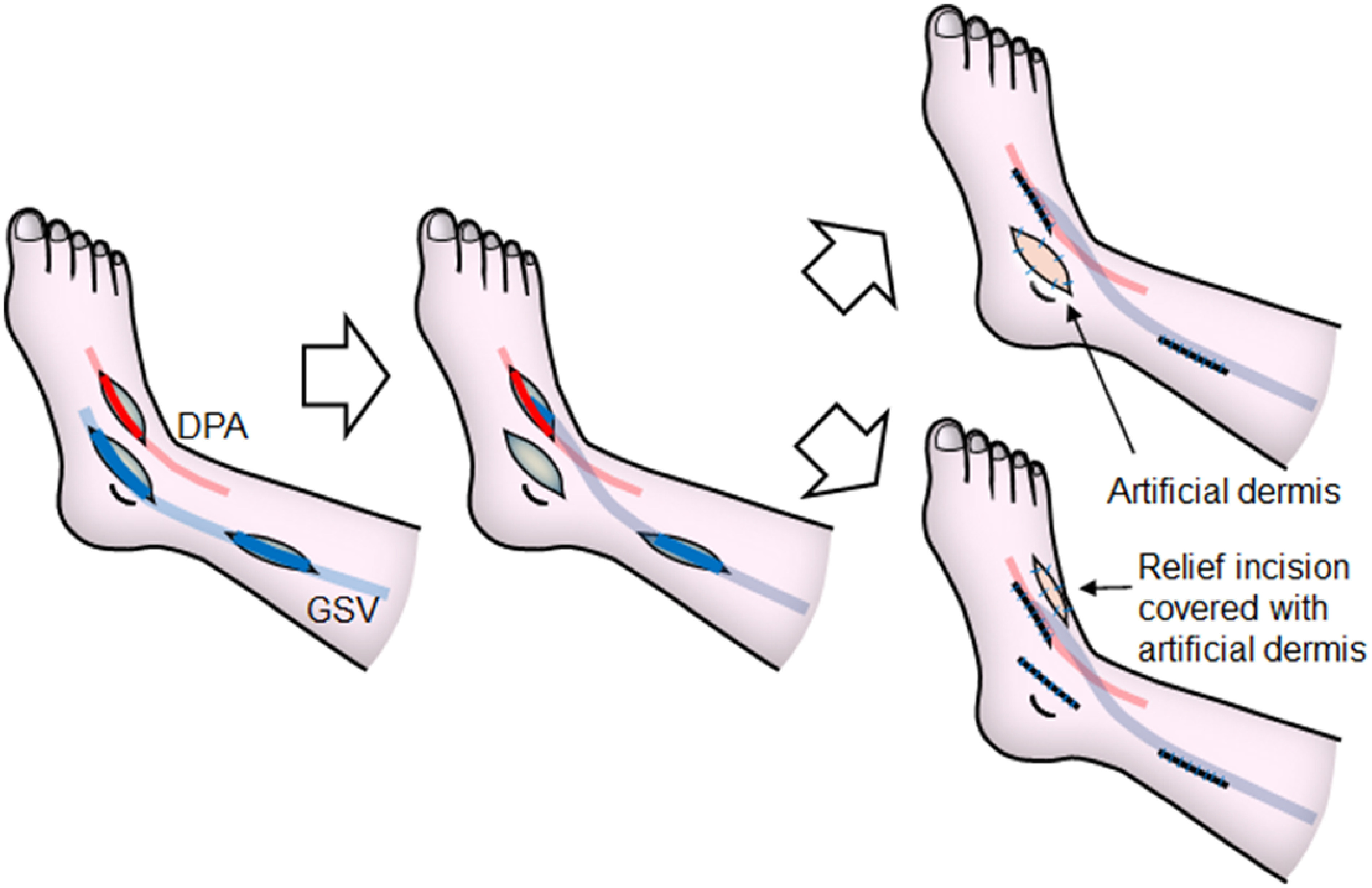
Fig. 2 Schema of the tension-free wound management in the bypass to the dorsalis pedis artery.

GSV: great saphenous vein; DPA: dorsalis pedis artery

Tension-free wound management was chosen to close the paramalleolar surgical wounds of distal bypass when excessive skin tension was recognized in order to prevent wound dehiscence. The surgical incision for the distal anastomotic site of the bypass was closed tension-free by interrupted subcutaneous sutures using absorbable threads and interrupted cutaneous sutures using nonabsorbable threads. On the contrary, the incisions for the SVG harvest were left open. When wound tension could not be reduced only by wound opening for the SVG harvest, an appropriate-sized relief incision was made at the suitable site to reduce the tension of the skin incision on the distal anastomosis. The opened incisions were covered with artificial dermis (Pelnac, Gunze Limited, Japan or Terudermis, Terumo Corporation, Japan). The technical methods of tension-free wound management in the paramalleolar bypass to the posterior tibial artery and dorsalis pedis artery are presented in **Figs. 1** and **2**, respectively.

From January 2010 to December 2018, 90 patients received distal bypass for CLTI. Among them, tension-free wound management, as described above, was adopted in one female and seven male patients (mean age, 64 years). Comorbidities of hypertension (n=3 patients), diabetes mellitus (n=7), hemodialysis (n=4), a history of coronary artery disease (n=4), and a history of cerebrovascular disease (n=2) were noted in the included patients. The Rutherford system was used to classify the severity of ischemic ulcers. Seven patients had a stage-5 Rutherford and one patient stage-6 Rutherford. The bypass target arteries were the dorsalis pedis artery in five patients and the posterior tibial artery in three patients. The bypass grafts used were reversed SVG in three patients and non-reversed SVG in five. Open wound management with artificial dermis was performed at the SVG harvest incision in seven patients and a relief incision in one patient.

Antiplatelet regimens, such as aspirin, cilostazol, or sarprogrelate hydrochloride, were prescribed for all patients throughout the follow-up period. Clinical assessment and duplex ultrasonographic analysis were performed at discharge and every 3 months thereafter. The study endpoints were graft patency, limb salvage, survival, and wound healing. Major amputation was defined as limb loss above the ankle level, and limb salvage was defined as freedom from major amputation.

The study was approved by the ethical committee of our institute (approval number: ER-202001). Written informed consent was obtained from all patients by comprehensive agreement method.

## Results

**Figure 3:**
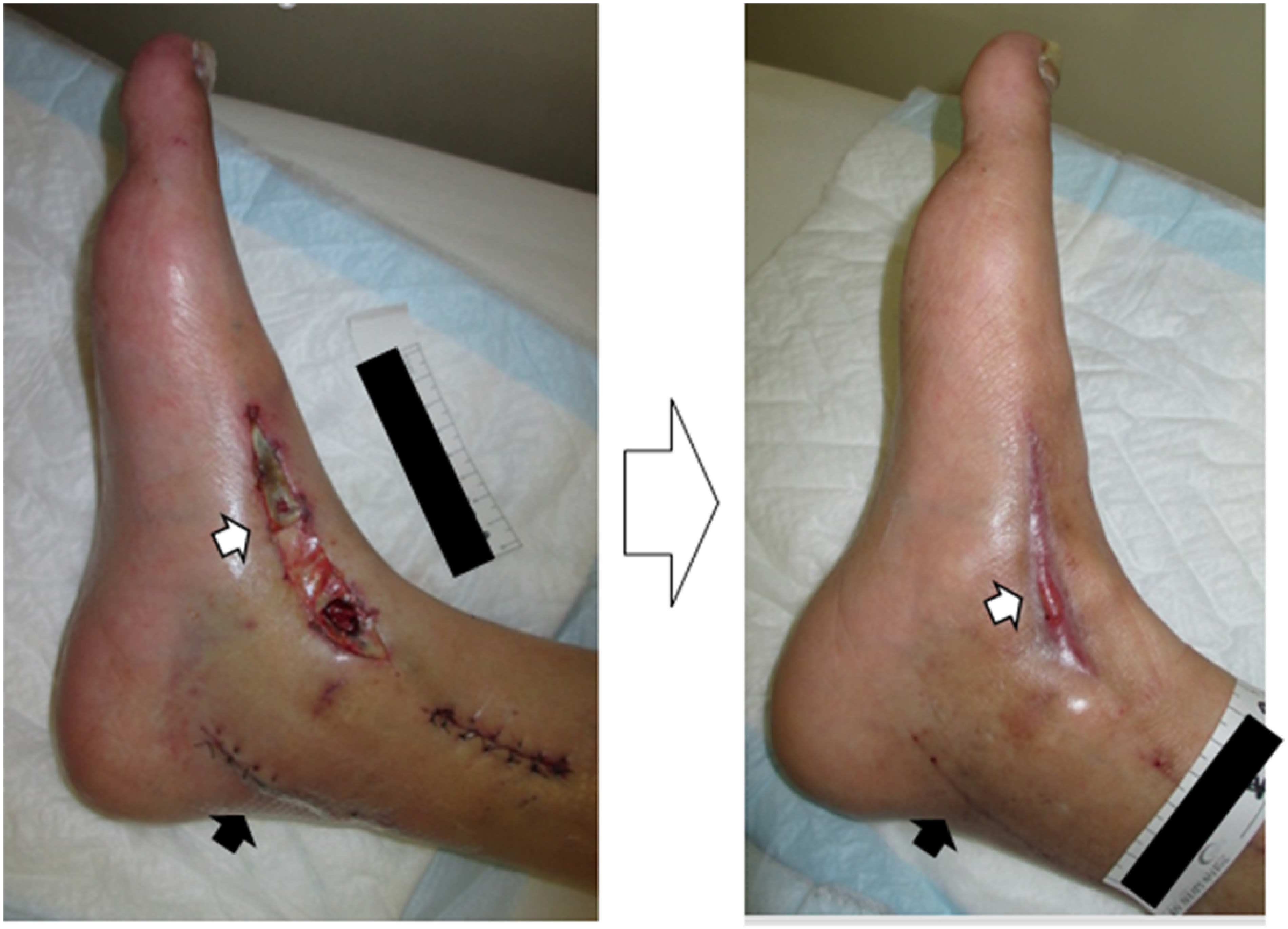
Fig. 3 Healing course of the wounds after bypass to the posterior tibial artery (a 73-year-old male patient). Black arrows indicate the surgical wound on the distal anastomosis and white arrows indicate the wound for saphenous vein graft harvest covered with artificial dermis. Left: 4 days after bypass, Right: 6 weeks after bypass.

**Figure 4:**
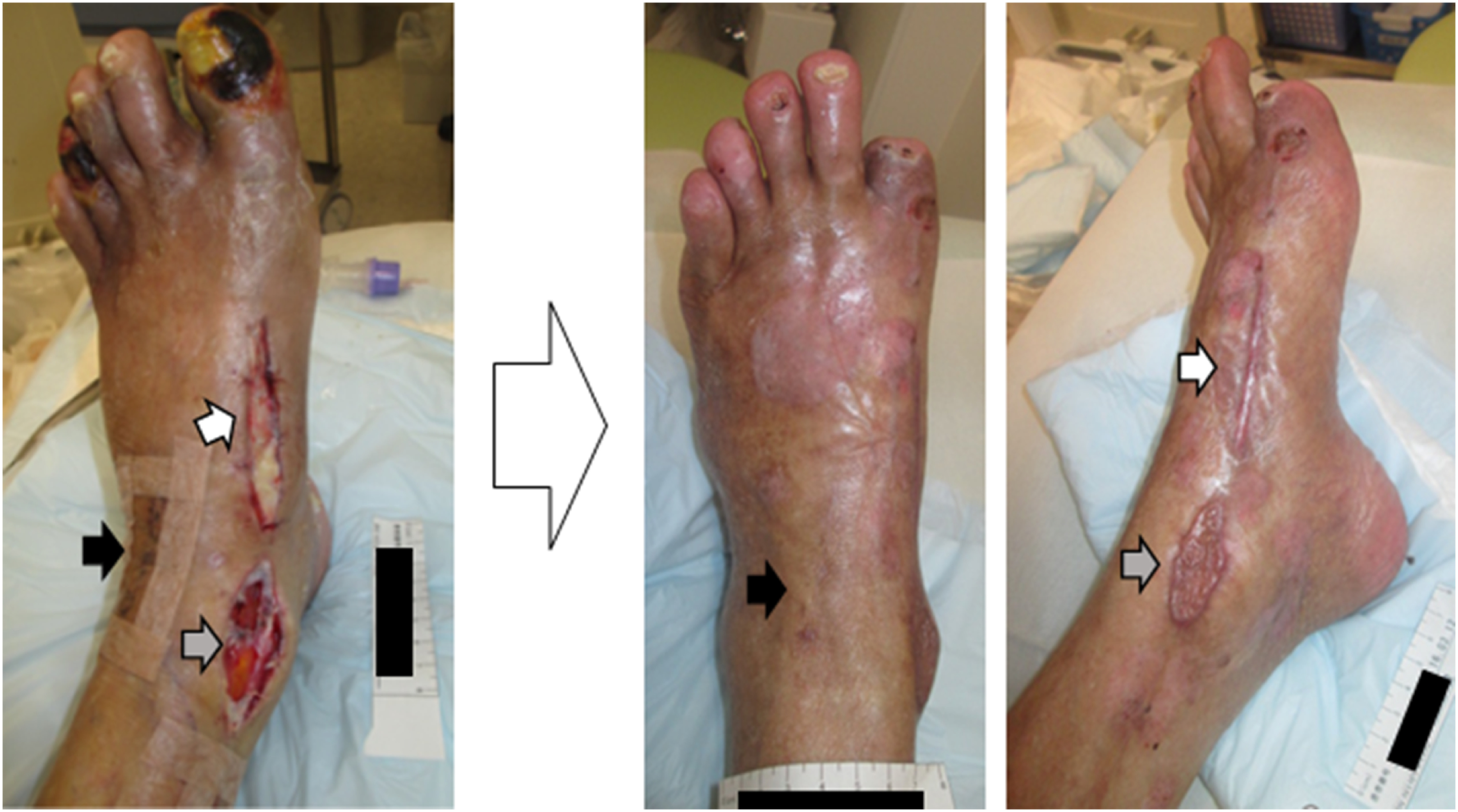
Fig. 4 Healing course of the wounds after bypass to the dorsalis pedis artery (a 60-year-old male patient). Black arrows indicate the surgical wound on the distal anastomosis, white arrows indicate the wound for saphenous vein graft (SVG) harvest covered with artificial dermis, and gray arrows indicate the wound for SVG harvest covered with artificial dermis and received skin transplantation. Left: 8 days after bypass, Right: 4 months after bypass.

The postoperative course was unremarkable in all patients. Within 1.8 months (1–4 months) after the bypass, all surgical incisions, including the distal anastomotic site, healed without infection or opening (**Figs. 3** and **4**). Skin transplantation was performed in two patients under general anesthesia concomitant with ischemic wound debridement, because the size of their unclosed wounds was large and it seemed to take time for complete epithelization. Those surgical wounds also healed without complications. Minor amputation was necessary in seven of the eight ischemic ulcers (toe amputation, 7; trans-metatarsal amputation, 1), but there was no major amputation. Two postoperative graft stenoses occurred at 3 and 12 months after the bypass, which were successfully rescued by additional endovascular intervention.

Secondary graft patency, wound healing, and limb salvage rates were 100% during an average follow-up period of 30 months (2–51 months). Two of the patients died of cardiac events at 31 and 41 months, respectively, after the bypass.

## Discussion

Paramalleolar bypass using SVG is a standard procedure for managing CLTI. The reported patency and limb salvage rates of this bypass are satisfactory.^1–5)^ However, patients with CLTI are at a high risk for various wound complications, such as surgical site infection, lymphorrhea, wound dehiscence, graft exposure, and subsequent graft failure.^1–19)^

Wengrovitz et al. investigated wound complications in 163 infrainguinal bypasses.^13)^ Severe wound complications with wound dehiscence and graft exposure occurred in 12 of them, which led to 4 major amputations and 1 death. Ouriel et al. reviewed the outcomes of 16 patients with exposed SVG.^14)^ All patients were initially treated conservatively with moist sterile dressings, followed by split-thickness skin graft coverage of the wounds after cleaning. The wounds healed in seven patients, whereas nine patients developed complications of hemorrhage and graft thrombosis. Recent clinical studies have demonstrated that infection-related graft exposures after lower extremity bypass were successfully treated using autologous tissue, such as pedicle muscle flaps.^15–17)^ This aggressive management of infected wounds might be effective, but involves increased length of hospitalization and cost.

Before the start of this clinical study, we encountered two patients with distal anastomotic rupture of the bypass to the dorsalis pedis artery following wound infection and dehiscence. One of them was a 58-year-old female patient with diabetes and stage-5 Rutherford ulcer. The anastomotic disruption with wound dehiscence occurred 3 weeks after popliteal-to-dorsalis pedis artery with non-reversed SVG. The other patient was a 78-year-old woman with diabetes and stage-5 Rutherford ulcer. The anastomotic rupture occurred 2 weeks after popliteal-to-dorsalis pedis artery with non-reversed SVG. Both patent SVGs had to be ligated and major amputation was inevitable in both patients, and we realized the importance of preventing wound dehiscence and decided to begin tension-free wound management. This wound management was adopted for selected patients in whom the skin tension was excessive for primary closure of paramalleolar surgical wounds; patients with several parallel skin incisions around the ankle for exposure of distal anastomotic site and the SVG harvest; patients with skin sclerosis by persistent ischemia or recurrent infection; and patients with inflammatory edema around the ankle. This method might be particularly effective for bypass to the dorsalis pedis artery which lies in the dorsum pedis with poor skin laxity and runs more peripheral than the posterior tibial artery.

There are a few studies of inventions to relieve skin tension in paramalleolar bypass for preventing dehiscence of distal anastomotic wounds. Mouton et al. reported the surgical method of a proximalized lateral tunnel for the bypass to the dorsalis pedis artery to protect the SVG exposure induced by wound breakdown.^18)^ Robison et al. introduced the ancillary technique (“pie-crusting”) to reduce skin tension in selected patients with pedal edema or difficult wound closure.^19)^ Multiple 4- to 5-mm stab incisions through the epidermis and dermis were made using a blade scalpel to facilitate wound closure. There were no distal wound problems among 15 patients who received this surgical technique and were considered to be at the greatest risk for wound breakdown.

In our tension-free wound management of paramalleolar bypass, an artificial dermis was used to cover the incision made for SVG harvest or the relief incision. Dermal regeneration templates, such as Pelnac or Terdermis, were designed to treat extensive burn injuries. To date, these techniques have been widely applied to manage various acute and chronic wound sites, and several clinical experiences of using these techniques have been reported in the field of plastic surgery.^20–23)^ In patients in whom artificial dermis is used for unclosed wounds, complete epithelization occurs in 4–8 weeks. Additional skin transplantation is occasionally needed; however, skin transplantation can be performed simultaneously with minor amputation or wound debridement, which are necessary in most patients with CLTI having ischemic ulcers or gangrene.

## Conclusion

Tension-free management of surgical wound using artificial dermis seems to be effective in selective patients who receive paramalleolar bypass for CLTI.

## Additional Note

The content of this study was partly presented at the 33rd Annual Meeting of European Society for Vascular Surgery on October 24–27, 2019, in Hamburg, Germany.

## References

[1] Bergamini TM, George SM Jr, Massey HT, et al. Intensive surveillance of femoropopliteal-tibial autogenous vein bypasses improves long-term graft patency and limb salvage. Ann Surg 1995; 221: 507-16. 7748032 10.1097/00000658-199505000-00008 PMC1234628

[2] Eckstein HH, Schumacher H, Maeder N, et al. Pedal bypass for limb-threatening ischaemia: an 11-year review. Br J Surg 1996; 83: 1554-7. 9014672 10.1002/bjs.1800831120

[3] Slim H, Tiwari A, Ahmed A, et al. Distal versus ultradistalbypass grafts: amputation-free survival and patency rates in patients with critical leg ischeamia. Eur J Vasc Endovasc Surg 2011; 42: 83-8. 21514854 10.1016/j.ejvs.2011.03.016

[4] Azuma N, Uchida H, Kokubo T, et al. Factors influencing wound healing of critical ischaemic foot after bypass surgery: is the angiosome important in selecting bypass target artery? Eur J Vasc Endovasc Surg 2012; 43: 322-8. 22237509 10.1016/j.ejvs.2011.12.001

[5] Shirasu T, Hoshina K, Nishiyama A, et al. Favorable outcomes of very elderly patients with critical limb ischemia who undergo distal bypass surgery. J Vasc Surg 2016; 63: 377-84. 26482994 10.1016/j.jvs.2015.08.090

[6] Schwartz ME, Harrington EB, Schanzer H. Wound complications after in situ bypass. J Vasc Surg 1988; 7: 802-7. 3373622 10.1067/mva.1988.avs0070802

[7] Harrington EB, Harrington ME, Schanzer H, et al. The dorsalis pedis bypass—moderate success in difficult situations. J Vasc Surg 1992; 15: 409-14; discussion, 415-6. 1735902 10.1067/mva.1992.33161

[8] Greenblatt DY, Rajamanickam V, Mell MW. Predictors of surgical site infection after open lower extremity revascularization. J Vasc Surg 2011; 54: 433-9. 21458203 10.1016/j.jvs.2011.01.034

[9] Kalish JA, Farber A, Homa K, et al. Factors associated with surgical site infection after lower extremity bypass in the Society for Vascular Surgery (SVS) Vascular Quality Initiative (VQI). J Vasc Surg 2014; 60: 1238-46. 24953898 10.1016/j.jvs.2014.05.012

[10] Smith AD, Hawkins AT, Schaumeier MJ, et al. Predictors of major amputation despite patent bypass grafts. J Vasc Surg 2016; 63: 1279-88. 26860641 10.1016/j.jvs.2015.10.101

[11] Davis FM, Sutzko DC, Grey SF, et al. Predictors of surgical site infection after open lower extremity revascularization. J Vasc Surg 2017; 65: 1769-78.e3. 28527931 10.1016/j.jvs.2016.11.053

[12] Aziz F, Bohr T, Lehman EB. Wound disruption after lower extremity bypass surgery is a predictor of subsequent development of wound infection. Ann Vasc Surg 2017; 43: 176-87. 28300677 10.1016/j.avsg.2016.10.065

[13] Wengrovitz M, Atnip RG, Gifford RR, et al. Wound complications of autogenous subcutaneous infrainguinal arterial bypass surgery: predisposing factors and management. J Vasc Surg 1990; 11: 156-63; discussion, 161-3. 2296096 10.1067/mva.1990.16918

[14] Ouriel K, Geary KJ, Green RM, et al. Fate of the exposed saphenous vein graft. Am J Surg 1990; 160: 148-50. 2200292 10.1016/s0002-9610(05)80295-8

[15] Calligaro KD, Veith FJ, Schwartz ML, et al. Management of infected lower extremity autologous vein grafts by selective graft preservation. Am J Surg 1992; 164: 291-4. 1415932 10.1016/s0002-9610(05)81090-6

[16] Reifsnyder T, Bandyk D, Seabrook G, et al. Wound complications of the in situ saphenous vein bypass technique. J Vasc Surg 1992; 15: 843-50; discussion, 848-50. 1578540 10.1067/mva.1992.36658

[17] Lermusiaux P, Laurent B, de Forges MR, et al. Infection-related exposure of the lower anastomosis of femorodistal bypass: salvage through use of pedicle muscle flaps. Ann Vasc Surg 2000; 14: 620-5. 11128457 10.1007/s100169910112

[18] Mouton WG, Otten KT, Fitridge RA. Proximalized lateral tunnel for the bypass to the dorsalis pedis artery—a safe way to go. Thorac Cardiovasc Surg 2001; 49: 245-6. 11505326 10.1055/s-2001-16105

[19] Robison JG, Ross JP, Brothers TE, et al. Distal wound complications following pedal bypass: analysis of risk factors. Ann Vasc Surg 1995; 9: 53-9. 7703063 10.1007/BF02015317

[20] Tchero H, Herlin C, Bekara F, et al. Failure rates of artificial dermis products in treatment of diabetic foot ulcer: a systematic review and network meta-analysis. Wound Repair Regen 2017; 25: 691-6. 28597935 10.1111/wrr.12554

[21] Lou X, Xue H, Li G, et al. One-stage Pelnac reconstruction in full-thickness skin defects with bone or tendon exposure. Plast Reconstr Surg Glob Open 2018; 6: e1709. 29707464 10.1097/GOX.0000000000001709 PMC5908513

[22] Park KS, Lee WS, Ji SY, et al. The treatment of post-traumatic facial skin defect with artificial dermis. Arch Craniofac Surg 2018; 19: 35-40. 29609430 10.7181/acfs.2018.19.1.35 PMC5894543

[23] Scuderi N, Fioramonti P, Fanelli B, et al. The use of dermal regeneration template (Pelnac®) in a complex upper limb trauma: the first Italian case report. Eur Rev Med Pharmacol Sci 2019; 23: 5531-4. 31298303 10.26355/eurrev_201907_18285

